# A genetic approach to identify amino acids in human GCN1 required for mediating Gcn2 activation

**DOI:** 10.17912/micropub.biology.002224

**Published:** 2026-07-27

**Authors:** Aditi Ghuge, Susanne Gottfried, Anja H Schiemann, Evelyn Sattlegger

**Affiliations:** 1 School of Food Technology and Natural Sciences, Massey University, Palmerston North, New Zealand; 2 Maurice Wilkins Centre for Molecular BioDiscovery, Massey University, Palmerston North, New Zealand

## Abstract

The conserved protein kinase GCN2 orchestrates cellular adaptation to amino acid starvation and other stress. Activation of GCN2 requires direct binding to its effector protein GCN1, via the RWDBD region in GCN1. In yeast, overexpression of the RWDBD, with or without the C-terminus (CTD), elicits a dominant-negative phenotype due to impairing Gcn1-Gcn2 interaction. Here we show that overexpressed human RWDBD+CTD also causes a dominant-negative phenotype in yeast, in a manner dependent on specific amino acids. All but one amino acid is conserved from yeast to human, suggesting conservation of the GCN2 binding parameters in GCN1, with minor evolutionary divergence.

**Figure 1. Overexpression of the human RWDBD+CTD elicits a dominant negative phenotype under amino acid starvation conditions in yeast:** **(A)** Yeast *gcn1Δ* strain H2556 overexpressing from a galactose inducible promotor GST alone, and isogenic wild-type strain H1511 overexpressing GST alone, the GST-tagged yeast RWDBD (ScRWDBD) or human RWDBD+CTD (HsRWDBD+CTD), with or without an amino acid substitution as indicated, were grown to exponential phase, and subjected to 10-fold serial dilutions. Five µL of each undiluted and of diluted cultures were transferred to solid medium containing glucose (control) or galactose (Gal) as carbon source, and 3-amino-1,2,4-triazole (3AT) as indicated, and incubated at 30°C. Representative images of at least 4 biological replicates are shown. **(B, C)** Yeast *gcn1Δ* strain H2556 and wild-type strain H1511 overexpressing GST alone, or GST-tagged HsRWDBD+CTD variants as indicated, were subjected to SQGAs as done in (A). Representative images of at least 4 biological replicates are shown. **(D)** The growth of strains in (B) (left side of the graph) and (C) (right side of the graph) was quantified from at least 4 biological replicates, and shown in a bar graph relative to that of the wild-type strain overexpressing HsRWDBD+CTD. Values from HsRWDBD+CTD variants that are significantly different to that of wild-type HsRWDBD+CTD are indicated with an asterisk (student's t-test, p<0.05). **(E)** Strains from (B) and (C) were grown to exponential phase, harvested, and the generated whole cell extracts subjected to western blotting using antibodies against the GST-tag present at the N-terminus for all overexpressed proteins, and against Pgk1 as reference for equal loading. A representative image of at least 4 biological replicates is shown. All samples were run on the same gel. **(F)** Using ImageJ (Schneider et al., 2012) the signal intensity of the GST-tagged proteins in E was quantified relative to that of Pgk1, and relative to the GST/Pgk1 ratio of HsRWDBD+CTD, and the values plotted in a graph. The values shown are the average of at least 4 biological replicates, and the standard error is shown. None of the values are significantly different (student's t-test, p<0.05). **(G)** Gcn1 proteins from *Saccharomyces cerevisiae* (referred to as yeast in this work), *Schizosaccharomyces pombe* , *Mus musculus* , and *Homo sapiens* (accession numbers P33892, Q10105, AAI50736.1, NP_006827.1) were aligned using Clustal Omega (Madeira et al., 2019). The displayed section of the alignment shows the region surrounding yeast Gcn1 Arg-2259. The predicted secondary structure of *S. cerevisiae* Gcn1, as modelled previously (Rakesh et al., 2017; Sattlegger and Hinnebusch, 2000), is shown beneath the ScGcn1 sequence. Helices predicted to interact with Gcn2 are indicated in blue. Gcn1 Arg-2259 shown previously to hamper Gcn1-Gcn2 interaction and to dampen Gcn2 activation is highlighted in pink (Gottfried et al., 2022; Sattlegger and Hinnebusch, 2000), and so are the equivalent amino acids in the other GCN1 proteins. All amino acids highlighted in the ScGcn1 sequence were shown previously to the be required for the ScRWDBD to elicit a dominant negative phenotype on starvation (3AT) medium (Gottfried et al., 2022; Sattlegger and Hinnebusch, 2000), and equivalent amino acids are highlighted with the same colour in the other GCN1 sequences.

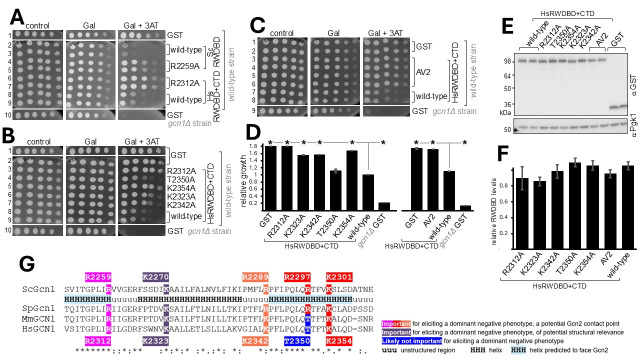



## Description

The protein kinase General control non-derepressible 2 (Gcn2 in *Saccharomyces cerevisiae* (yeast) and GCN2 in mammals; for simplicity we use the mammalian nomenclature throughout the manuscript body) is a key component of a conserved eukaryotic signalling pathway that adjusts protein synthesis to cellular needs (Hinnebusch 2005). Upon detection of amino acid shortage, GCN2 activation ultimately leads to a shift in the cell’s gene expression profile to promote adaptation to the adverse condition (Hinnebusch 2005).

Beyond amino acid homeostasis, GCN2 contributes to memory formation, feeding behaviour, and immune regulation, to just name a few (Castilho et al. 2014). For all roles investigated thus far, GCN2 requires its effector protein GCN1 for function (Castilho et al. 2014). Even though GCN2 is implicated in fundamental biological functions, as well as in diseases such as cancer (Castilho et al. 2014, Prescott et al. 2025), the molecular mechanisms underlying GCN2 activation is far from being understood. So far it is known that for activation, GCN2 must directly bind GCN1 via its RWD (a motif found in R ING finger-, WD -repeat-, and yeast DEAD (DEXD)-like helicase proteins) domain (Kubota et al. 2000, Sattlegger and Hinnebusch 2000). In yeast GCN1, the minimal region sufficient for GCN2 binding was dubbed as the RWD binding domain (RWDBD, though more accurately described as a region since it is not a discrete structural domain), with Arg-2259 in this region being critical for GCN2 binding (Sattlegger and Hinnebusch 2000, Rakesh et al. 2017). In mammals, studies also suggest that the RWDBD is required for GCN2 activation (Yamazaki et al. 2020), and that GCN1-GCN2 interaction is important for GCN2 activation (Silva et al. 2016).

In mammals, overexpressed RWDBD is sufficient to hamper the GCN2 signalling pathway (Cambiaghi 2014). Similarly, in *S. cerevisiae* , overexpressed RWDBD disrupts GCN1-GCN2 interaction, reduces GCN2 activation, and this is associated with a reduced ability to grow under starvation conditions (Sattlegger and Hinnebusch 2000). Hence, this dominant-negative phenotype serves as an indicator for RWDBD-GCN2 interaction. Taking advantage of this dominant-negative phenotype, additional amino acids had been identified in *S. cerevisiae* RWDBD that are required for GCN2 activation, strongly suggesting that these as well are required for GCN2-binding (Gottfried et al. 2022) (Fig. 1G).

Here we aimed to determine the extent to which the amino acids in GCN1 required for GCN2 activation are conserved between yeast and humans. By analogy to the dominant-negative phenotype caused by overexpression of the *S. cerevisiae* RWDBD (ScRWDBD), we overexpressed the human equivalent in yeast, from a plasmid and a galactose inducible promotor. Though, we used HsRWDBD that included the HsGCN1 CTD, since the HsRWDBD alone was unable to elicit a dominant negative phenotype, likely due to its insufficient expression levels. The plasmids expressing ScRWDBD and HsRWDBD+CTD, respectively, were transformed into wild-type yeast strain H1511, and the transformants subjected to semi-quantitative growth assays (SQGAs) using solid medium containing 3-amino-1,2,4-triazole (3AT), a compound that induces histidine starvation (Hilton et al. 1965). Images of the plates are shown from the incubation day on which growth differences could be observed most clearly, if present. On galactose plates, all transformants exhibited slower growth compared with those on control plates containing glucose. This is a common phenomenon, as glucose is a more efficient carbon source than galactose. As found previously for ScRWDBD (Sattlegger and Hinnebusch 2000), galactose induced HsRWDBD+CTD overexpression leads to impaired growth on 3AT, indicative of hampered GCN1-GCN2 interaction (Fig. 1A, rows 1 vs 2&3, 1 vs 8&9). This suggests that HsRWDBD+CTD is capable of hampering endogenous GCN1-GCN2-interaction in yeast. We did note that the dominant negative phenotype elicited by ScRWDBD appeared to be stronger than that elicited by HsRWDBD+CTD. We cannot exclude the possibility that this is due to differences in expression levels. Nevertheless, of particular importance to this study is the fact that HsRWDBD+CTD can elicit a dominant negative phenotype. In HsRWDBD+CTD, Ala substitution of Arg-2312 (equivalent to yeast Arg-2259), reversed the dominant negative effect, as found previously for the R2259A substitution in ScRWDBD (Fig. 1A, rows 6&7 vs 8&9, and 4&5 vs 2&3) (Sattlegger and Hinnebusch 2000). Given that the SQGA uses 10-fold serially diluted cultures, it appears that the growth difference between the strains expressing the wildtype and the mutated protein is 100-fold, for the human as well as the yeast protein (Fig. 1A, rows 6&7 vs 8&9, and 4&5 vs 2&3). This may suggest that this Arg residue in human and yeast are equally relevant for GCN2 binding.

To identify additional amino acids in human GCN1 that are equivalent to the ones found in yeast to be important to elicit a dominant negative phenotype, a multisequence alignment was performed ( Fig. 1G ). In ScRWDBD so far a total of 5 amino acids were identified previously to be required for GCN2 inhibition. While four likely engage in direct GCN2 binding, the fifth is relevant for ensuring the proper orientation of the two helixes involved in direct GCN2 binding (Fig. 1G) (Sattlegger and Hinnebusch 2000, Gottfried et al. 2022). In HsRWDBD+CTD, these equivalent five amino acids were individually subjected to Ala substitutions, the resulting HsRWDBD+CTD variants introduced into yeast, followed by SQGAs. We found that the R2312A and K2354A substitutions each reverted the dominant-negative phenotype elicited by HsRWDBD+CTD, while the K2323A and K2342A substitutions each reverted the dominant-negative phenotype in part (Fig 1B,D). This suggests that these amino acids are required, in full or in part, for binding GCN2. On the other hand, while R2297A substitution reverted the dominant-negative phenotype of ScRWDBD (Gottfried et al. 2022), T2350A substitution in HsRWDBD+CTD did not. This would suggest that in HsRWDBD this GCN2 contact point does not exist. We did verify that the substitutions did not affect the HsRWDBD+CTD protein levels, by performing immunoblotting assays, using antibodies against the GST-tag present in all HsRWDBD+CTD variants, and against Pgk1 as loading control (Fig. 1E,F).

Simultaneous R2312A, K2342A, T2350A, and K2354A substitutions in AV2 appeared to fully revert the dominant-negative phenotype (Fig. 1C), as expected, since some individual substitutions alone were sufficient to fully reverse the phenotype (Fig. 1B). The fact that mutations in the RWDBD are capable of reverting the phenotype elicited by HsRWDBD+CTD overexpression is in the agreement with the idea that the RWDBD in human GCN1 is required for GCN2-GCN1 interaction as found for the yeast RWDBD. 

In conclusion, of the 5 amino acids investigated for their role in GCN2 activation, four are conserved between yeast and humans (in humans: R2312, K2323, K2342, K2354), while one was not (T2350). This may indicate that minor evolutionary divergence has occurred within the GCN2 interaction interface of GCN1. We cannot exclude the possibility that this divergence may concurrently have led to divergence in the HsGCN2 RWD. Nevertheless, given that HsRWDBD+CTD was able to elicit a dominant-negative phenotype in yeast, this strongly suggests that the conserved GCN2 contact points in GCN1 are sufficient for mediating interspecies RWDBD-GCN2 interaction. Along the same line, it is tempting to speculate that major GCN1 contact points in GCN2 should be highly conserved as well from yeast to human.

## Methods

**Yeast strains and plasmids used**

Yeast strains and plasmids used in this study are provided in Tables I and II . Plasmids were generated commercially via site-directed mutagenesis (Genscript, USA). The vector used was pES128-9 (Sattlegger and Hinnebusch 2000).

**Semi-quantitative growth assay (SQGA)**

SQGAs were carried out as described previously (Ghuge et al. 2023). Briefly, overnight cultures were serially diluted tenfold and spotted onto solid synthetic medium, with 60 mM 3AT added when indicated. The strains were then incubated at 30°C, and growth was recorded using a flatbed scanner.

**Generation of whole cell extracts and Western blotting**

Whole cell extracts were generated from exponentially growing cells, aliquots subjected to denaturing polyacrylamide gel electrophoresis, and then subjected to Western blotting using primary antibodies as indicated in Figure 1 (Table III), as published previously (Lee et al. 2017; Anderson and Sattlegger 2021). Bound antibodies were detected with horseradish peroxidase-conjugated secondary antibodies, using Pierce ECL Western Blotting Substrate (#32209, Thermo Scientific, USA) and the ChemiDoc™ Imaging System (Bio-Rad, USA).

## Reagents

**Table I: Strains**

**Table:** 

**strain**	**genotype**	**source**
H1511	* MATα ura3-52 trp1-63 leu2-3,112, GAL2 ^+^*	(Foiani et al. 1991)
H2556	as H1511 but *gcn1Δ*	(Sattlegger and Hinnebusch 2000)

**Table II: Plasmids**

**Table:** 

**plasmid**	**Protein**	**marker**	**vector**	**source**
pES124-B2	GST-ScRWDBD [ScGcn1 amino acids 2052–2428]	* Amp ^R^ , URA3, leu2Δ *	pES128-9	(Sattlegger and Hinnebusch 2000)
pES167-2E	as pES124-B2 but R2259A	* Amp ^R^ , URA3, leu2Δ *	pES128-9	(Sattlegger and Hinnebusch 2000)
pES503-WT	GST‐HsRWDBD+CTD-myc [HsGCN1 amino acids 2093-2671]	* Amp ^R^ , URA3, leu2Δ *	pES128-9	This work
pSG38/AS10	as pES503-WT but R2312A	* Amp ^R^ , URA3, leu2Δ *	pES128-9	This work
pES503-K2323A	as pES503-WT but K2323A	* Amp ^R^ , URA3, leu2Δ *	pES128-9	This work
pES503-K2342A	as pES503-WT but K2342A	* Amp ^R^ , URA3, leu2Δ *	pES128-9	This work
pES503-T2350A	as pES503-WT but T2350A	* Amp ^R^ , URA3, leu2Δ *	pES128-9	This work
pES503-K2354A	as pES503-WT but K2354A	* Amp ^R^ , URA3, leu2Δ *	pES128-9	This work
pES503-AV2	as pES503-WT but R2312A, K2342A, T2350A, K2354A	* Amp ^R^ , URA3, leu2Δ *	pES128-9	This work

**Table III: Antibodies**

**Table:** 

**Antibody**	** Antibody type**	**Dilution**	**Order number, Source**
anti GST	rabbit polyconal antibodies	1:5,000	#SC-459, Santa Cruz, USA
anti Pgk1	mouse monoclonal antibodies	1:5,000	#459250, Thermo Scientific, USA
anti rabbit	horseradish peroxidase-conjugated donkey antibodies	1:50,000	#31458, Thermo Scientific, USA
anti mouse	horseradish peroxidase-conjugated goat antibodies	1:50,000	#31430, Thermo Scientific, USA
